# Predictive value of C-reactive protein levels for the early and later detection of postoperative complications after cytoreductive surgery and HIPEC

**DOI:** 10.3389/fonc.2022.943522

**Published:** 2022-10-25

**Authors:** Alexia Roux, Valentin David, Sylvia Bardet M, Emilie Auditeau, Sylvaine Durand Fontanier, Abdelkader Taibi

**Affiliations:** ^1^ Visceral Surgery Department, Limoges University Hospital, Limoges, France; ^2^ University Limoges, Limoges, France; ^3^ University Limoges, Centre National de la Recherche Scientifique (CNRS), Multidisciplinary Research Institute (XLIM), Limoges, France

**Keywords:** postoperative complications, C-reactive protein, peritoneal metastasis, non-infective complications, infective complications, HIPEC, cytoreductive surgery

## Abstract

**Synopsis:**

C-reactive protein (CRP), white blood cells and procalcitonin (PCT) participate in the systemic response to inflammation and increase after postoperative infective complications. Postoperative complications after CRS and HIPEC could be predicted using the CRP cut-off value (169 mg/L at PODs 3-5 and 62 mg/L at PODs 7-10).

**Background:**

Postoperative elevation of C-reactive protein (CRP) can be used in order to predict the postoperative complications in many indications. Cytoreduction surgery (CRS) associated with hyperthermic intraperitoneal chemotherapy (HIPEC) is associated with high morbidity.

**Objectives:**

The aim of the study was to demonstrate the CRP predictive value for the occurrence of complications.

**Methods:**

All patients who had CRS and HIPEC, regardless of the origin of peritoneal metastasis, were included in this retrospective study. Postoperative complications and CRP and white blood cell (WBC) counts were recorded from postoperative day (POD) 1 through 10.

**Results:**

Among the 127 patients included, 58 (45.7%) had no complications (NCs), 53 (41.7%) had infective complications (ICs), and 16 (12.6%) had non-infective complications (NICs). The IC group had a higher CRP value than the NC group, which was statistically significant from POD7 to POD10 (41.1 versus 107.5 p = 0.023 and 77.8 versus 140 p = 0.047, respectively). A cut-off CRP value was 169 mg/L at PODs 3-5 and 62 mg/L at PODs 7-10. The area under the curve (AUC) at POD5 was 0.56 versus 0.76 at POD7, p=0.007. The sensibility, specificity, positive and negative predictive values of these cut-offs were 55%, 83%, 74% and 67%, respectively. Moreover, 17 patients (32%) with ICs had a CRP value higher than these cut-offs before the diagnosis was made by the medical team.

**Conclusion:**

This study suggested that postoperative complications could be predicted using the CRP cut-off value on PODs 3-5 (169 mg/l) and PODs 7-10 (62 mg/l) after CRS and HIPEC.

## Introduction

Over the past decade, cytoreduction surgery (CRS) associated with hyperthermic intraperitoneal chemotherapy (HIPEC) has been used to treat peritoneal metastasis (PM) originating from different tumours. Its usefulness is less proven in other types of digestive cancers and is discussed on a case-by-case basis in multidisciplinary oncological meeting for PM from gastric or biliary cancers (1, 2). It is a heavily skilled surgical procedure that can lead to complications secondary to surgery (anastomotic leakage, intra-abdominal abscesses) and chemotherapy (thrombocytopenia, haemorrhage), and the complication rate is estimated to be 24% at 2 months postoperatively (3).

C-reactive protein (CRP), white blood cells and procalcitonin (PCT) participate in the systemic response to inflammation and increase after postoperative infective complications (4). The usefulness of CRP as a marker of septic complications has been demonstrated by several authors (5, 6). Most of these studies have found a cut-off value of CRP at a concrete postoperative day (POD) that predicts postoperative complications, especially infective complications.

The use of CRP, PCT and white blood cell (WBC) in postoperative monitoring has been poorly assessed after HIPEC (7). Currently, no study has established a CRP « cut-off » that can lead the surgeon to search for postoperative complications, and the HIPEC procedure produces an inflammatory response in all patients undergoing cytoreductive surgery (8). The utility of a CRP cut-off value for predicting which patients are at greatest risk of complications following peritoneal metastasis surgery is an important topic that has not been evaluated and can help the peritoneal surgeons. The aim of this study was to evaluate the predictive value of CRP for detecting postoperative infectious complications following CRS and HIPEC and to establish clinically valuable cut-off values for CRP levels.

## Materials and methods

### Study population

All patients over 18 years of age who had undergone HIPEC associated with cytoreduction surgery at our university hospital, regardless of the origin of peritoneal metastases, were included between 01/2010 and 02/2020 in this retrospective study.

### Inclusion and exclusion criteria

Patients were selected after preoperative radiological examinations and all cases are discussed in a multidisciplinary oncology meeting. Only patients with limited resectable MP (i.e. with PCI < 15 for colorectal origin, ovarian origin or with resectable mesothelioma or pseudomyxoma) according to the French recommendations (9, 10), had a cytoreduction surgery and HIPEC. If the patient had extensive or non-resectable PM on the preoperative work-up including CT-Scan +- MRI +- PET Scan +- Laparoscopy, he received palliative treatment.

### Perioperative care and HIPEC procedure

All participants underwent a median laparotomy, and explorative laparotomy was performed first to evaluate the peritoneal cancer index (PCI). Complete, visible resection of all PMs, when needed, visceral resection, and multiorgan resection, e.g., the liver and spleen, were performed in order to achieve curative surgery. Then, HIPEC procedure was performed with Oxaliplatin, Mitomycin, Doxurubicin or Cisplatin.

### Initial data analysis

The following data were recorded: age, sex, body mass index (BMI), American Society of Anesthesiology (ASA) score, primary tumour site, surgical procedures (digestive resection, stoma, estimated blood loss), PCI, chemotherapy used during HIPEC, and CC score.

### Postoperative follow-up

All patients were followed up and examined at each visit, every day, by the surgeon and anaesthetist. If the patient had symptoms, the medical team performed a specific exam (urinary test, radiological exams) according to French guidelines (**Annex 2**).

Postoperative complications were recorded during 3 months. CRP level and the WBC count were recorded from postoperative day (POD) 1 through 10, as well as the mean length of stay (LOS) and mortality at 3 months.

### Definitions of complications

All patients were examined daily and were divided into three groups:

- without complications (no complications (NCs)),
- group with infective complications (ICs) according to Common Terminology Criteria for Adverse Events (CTCAE) Version 5.0 (11), including pneumonia, subcutaneous abscess, urinary tract infection, anastomotic leakage, intra-abdominal abscess and central venous catheter infection. These ICs were confirmed by clinical and radiologic or bacteriological exams.
- group with non-infective complications (NICs) to Common Terminology Criteria For Adverse Events (CTCAE) Version 5.0 (11), such as postoperative bleeding, digestive occlusion, respiratory failure, acute renal failure, thrombocytopenia, venous thrombosis, pulmonary embolism, and peripheral neuropathy. These NICs were confirmed by clinical and radiological or biological exams.

### Endpoints

The objective was to analyse the ability of CRP to predict ICs and NICs in the first 10 PODs.

To improve the comparison of tested values, we summarized the values of POD 3 and 5 (very early complications) and of POD 7 and 10 (later complications) and used the highest measured value. Moreover, we calculated the optimal cut-off values using ROC analysis.

The secondary endpoints were the incidence of postoperative ICs, according to the WBC levels. All procedures were in accordance with the Helsinki Declaration.

### Statistical analysis

Data are shown as means ± SD for quantitative variables or numbers and percentages for qualitative variables. The baseline data and the occurrence of endpoints were analyzed using the parametric t test or the nonparametric U test for continuous variables. The Chi-squared test or Fisher’s exact test was used for categorical variables as appropriate. An ANOVA parametric test was used as well, to compare between the two groups (No complication versus with ICs), and between the two others groups (No complication versus with NICs). A multivariate logistic regression analysis was further performed, confuting the PCI and the origin of PM as a confounder, affecting the CRP statistical correlation with infective complications. Statistical analysis was performed with GraphPad Prism v8.0. The cut-off value for the CRP ratio was determined using receiver operating characteristic (ROC) curves. The area under the curve and 95% confidence interval of the ROC curve were calculated using Stata 11. Values of p< 0.05 were considered statistically significant. To evaluate the predictive value of these cut-offs on the occurrence of complications, we also calculated the sensitivity, specificity, positive predictive value and negative predictive value.

## Results

### Patient characteristics and postoperative complications

A total of 166 patients were initially eligible for inclusion in the study. Of these, 5 patients received 2 HIPEC and were therefore included twice. Forty-four patients were excluded: 34 due to a lack of data (no CRP values collected in 10 days), 5 because surgical exploration did not allow curative management and received intravenous chemotherapy (high PCI, metastasis, local invasion), and 5 patients who had CRS without HIPEC. **Annex 1**

A total of 127 patients who had undergone HIPEC were included. **Table 1** The study population consisted of 88 women (69.3%) and 39 men (30.7%). All patients with colorectal and ovarian PMs received preoperative IV chemotherapy. Patients with primary peritoneal cancer received surgery treatment in front line.

**Table 1:** Patients characteristics and postoperative complications.

	No Complication	Infective complications	P (No complications group vs Infective complications group)	No infective complications	P (No complication group vs No infective complications group)
Characteristic	N = 58	N = 53		N = 16	
**Sex** (**n, %)**					
Male	16 (27,6%)	19 (35,8%)	*0,4*	4 (25%)	*1*
Female	42 (72,4%)	34 (64,2%)	*0,4*	12 (75%)	*1*
**Age (years) (mean-ranges)**	61,4 (37-74)	59,5 (29-77)	*0,3*	61,5 (36-75)	*0,9*
**BMI (kg/m2) (mean +- SD)**	24,7 (3,9)	25,3 (+-4,9)	*0,5*	26,1 (+-8,4)	*0,4*
**ASA Score**					
ASA 1-2	32 (55,2%)	32 (60,3(%)	0,7	7 (43,7%)	0,4
ASA 3-4	26 (44,8%)	21 (39,7%)	0,7	9 (56,3%)	0,4
**Origin of PM (n, %)**					
Colorectal	21 (36,2%)	31 (58,5%)	***0,02***	6 (37,5%)	*1*
Ovarian	23 (39,7%)	13 (24,5%)	*0,1*	6 (37,5%)	*1*
Peritoneum	12 (20,7%)	6 (11,3%)	*0,2*	4 (25%)	*0,7*
**Neoadjuvant chemotherapy (n, %)**	49 (71%)	48 (90,6%)	*0,4*	10 (62,5%)	*0,08*
**PCI (mean, +- SD)**	7 (+- 6.3)	8,9 (+-6.5)	*0,1*	9,1 (+- 6.1)	*0,4*
**HIPEC (n, %)**					
Oxaliplatine	35 (60,3%)	36 (67,9%)	*0,7*	10 (62,5%)	*0,9*
Mitomycine	16 (27,6%)	12 (22,6%)	*0,7*	5 (31,2%)	*0,8*
Cisplatine	7 (12,1%)	4 (7,6%)	*0,5*	1 (6,3%)	*0,7*
**CC score (n, %)**					
CC0	51 (87,9%)	41 (77,4%)	*0,2*	11 (68,8%)	*0.1*
CC1	4 (6,9%)	9 (16,9%)	*0,1*	3 (18,7%)	*0,2*
CC2	3 (5,2%)	3 (5,7%)	*1*	2 (12,5%)	*0,3*
**Operative procedure** (**n, %)**					
Resection and digestive anastomosis	19 (32,8%)	26 (49,1%)	*0,08*	11 (68,7%)	***0.01***
Digestive resection without anastomosis	9 (15,5%)	13 (24,5%)	*0,2*	1 (6,2%)	*0,7*
Gallbladder resection	47 (81%)	43 (73,6%)	*1*	9 (56,3%)	***0,05***
Omentectomy	47 (81%)	43 (73,6%)	*1*	11 (68,8%)	*0,3*
Liver resection or radiofrequency	5 (8,6%)	9 (17%)	*0,25*	1 (6,3%)	*1*
Diaphragm resection	1 (1,7%)	1 (1,9%)	*1*	0 (0%)	*0,4*
Total Peritonectomy	12 (20,7%)	6 (11,3%)	*0,2*	4 (25%)	*0,7*
Ovariectomy	15 (25,9)	18 (34%)	*0,4*	5 (31,3%)	*0,7*
Vaginal resection	2 (3,5%)	1 (1,9%)	*0,6*	1 (6,3%)	*0,5*
Hysterectomy	8 (13,8%)	6 (11,3%)	*0,7*	3 (18,8%)	*0,7*
Appendectomy	16 (27,6%)	7 (13,2%)	*0,1*	2 (12,5%)	*0,4*
Splenectomy	3 (5,2%)	0 (0%)	*0,1*	2 (12,5%)	*0,3*
Bladder resection	1 (1,7%)	1 (1,9%)	*1*	0 (0%)	*0,4*
**Estimated blood loss (ml)** **(mean +- SD)**	525 (+- 450)	478 (+- 301)	*0.3*	285 (+- 177)	*0.3*

ASA Score, American Society of Anesthesiologists; BMI, body mass index; CC score, completeness of Cytoreduction score; HIPEC, hyperthermic intraperitoneal chemotherapy; n, number; PCI, peritoneal cancer index; PM, peritoneal metastasis; SD, standard deviation.

Bold values = p values < 0.05.

Of the patients analyzed, the global morbidity rate was 54.3%: 45.7% (58 of 127) presented with no complications (NCs), 41.7% (53 of 127) had infective complications (ICs), and 12.6% (16 of 127) had non-infective complications (NICs). **Table 2**

**Table 2:** Postoperative complications according to the Common Terminology Criteria for Adverse Events (CTCAE) Version 5.0.

	Infective complications	No infective complications
**Any grade ≥2 adverse event n (%)**		
**Grade II n (%)**		
Pneumonia	3 (5,7%)	
Colitis	3 (5,7%)	
Urinary tract infection	**22 (41,4%)**	
Wound abscess	7 (13,2%)	
Infection of central venous catheter	6 (11,3%)	
Fever of Unknown Origin	6 (11,3%)	
Phlebitis		1 (6,2%)
Respiratory complication		**3 (18,9%)**
Acute kidney failure		**4 (25%)**
Others		4 (25%)
**Grade III n (%)**		
Anastomosis leakage	3 (5,7%)	
Intra abdominal abscess	3 (5,7%)	
small bowel obstruction		2 (12,5%)
Post operative bleeding		1 (6,2%)
**Grade IV n (%)**		
Pulmonary embolism		1 (6,2%)

Bold values = p values < 0.05.

**Table 1** presents the descriptive data of the 3 groups (NCs versus ICs and NCs versus NICs groups) and the perioperative data.

The length of hospital stay was significantly higher in the ICs group than in the NCs group (31 to 15.1 days [3.77; 10.89] p= 0.0001), whereas there was no significant difference between the NICs and the NCs groups (26 to 15.1 days [4.72; 26.80] p= 0.2). No patient died during the three postoperative months.

### CRP value in the three groups (NCs versus ICs and NCs versus NICs groups)

**Figure 1:**
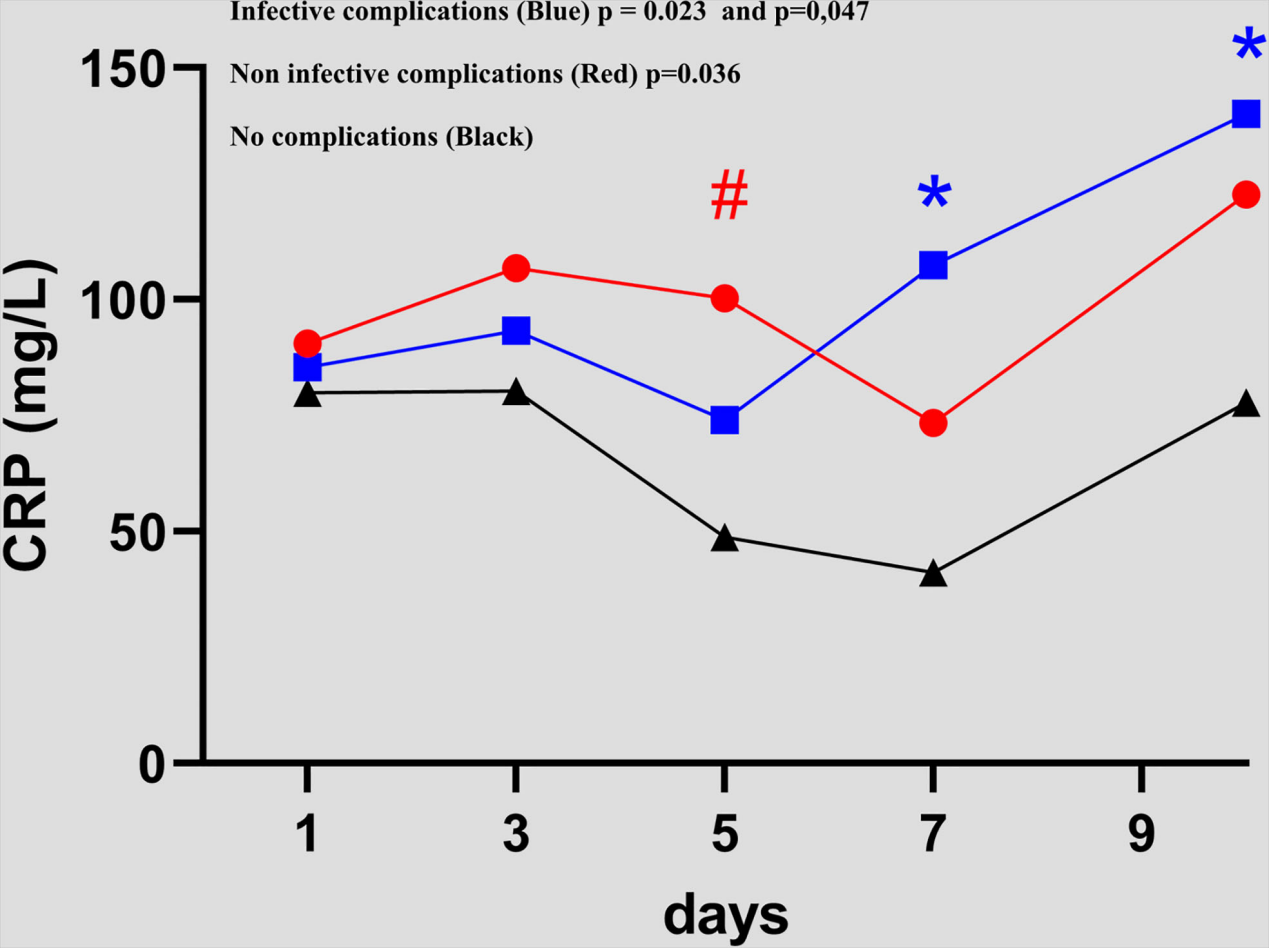
Evolution of C-reactive protein value between day 1 and day 10 in the 3 groups *(NCs*versus*ICs and NCs*versus*NICs groups).*. #, * = statistically significant.

ICs patients had a higher CRP value than NCs patients, which was statistically significant from POD 7 to POD 10 (41.1 versus 107.5 p = 0.023 and 77.8 versus 140 p = 0.047, respectively). **Figure 1** NICs patients had a higher CRP value than NCs patients on POD 5 (48.7 versus 100.3 p = 0.036). A CRP peak occurred during the 72 hours for the three groups.

In contrast to the NICs and NCs groups, in the ICs group, the CRP level increased progressively between POD 3 and POD 10. A progressive increase was observed in the NICs group at POD 10. The means and values are shown in **Table 3**. No significant difference between CRP values was found between the NCs versus ICs groups, and NCs versus NICs groups.

**Table 3:** Postoperative values of CRP and the WBC count in the 3 groups.

Variable	No complication (N = 58)	Infective complications (N = 53)		No Infective Complications (n = 16)	
	Mean +/- SD	Mean +/- SD	*P (No complication group*vs*Infective complications group)*	Mean +/- SD	*P (No complication group*vs*No Infective complications group)*
***CRP Value***					
POD 1	79,9 +/- 29,1	85,4 +/- 41	*0,5*	90,6 +/- 37,2	*0,3*
POD 3	80,3 +/- 60,8	93,3 +/- 80	*0,4*	106,8 +/- 67,9	*0,3*
POD 5	48,7 +/- 46,2	74,1 +/- 78,8	*0,2*	100,3 +/- 70,8	***0,04***
POD 7	41,1 +/- 40,8	107,5 +/- 104,1	***0,02***	73,4 +/- 45,8	*0,1*
POD 10	77,8 +/- 81,8	140 +/- 107,9	***0,047***	122,6 +/- 126,2	*0,3*
***WBC count***					
POD 1	11,1 +/- 3,3	11,2 +/- 4,2	*0,9*	11,5 +/- 5,5	*0,7*
POD 3	8,8 +/- 3,1	8,6 +/- 3	*0,7*	9,5 +/- 4,4	*0,5*
POD 5	7,9 +/- 2,5	8,6 +/- 3,6	*0,3*	8,1 +/- 2,6	*0,9*
POD 7	9,5 +/- 3,2	11,2 +/- 5,3	*0,08*	10 +/- 2,6	*0,6*
POD 10	9,1 +/- 3	11,9 +/- 6,1	***0,008***	10 +/- 2,7	*0,4*
***Length of stay (mean)***	15,1	31	***0,0001***	26,1	*0,2*
***Mortality < 3 months***	0	0	1	0	*1*

CRP, c reactive protein; POD, postoperative day; WBC, white bloods cells.

Bold values = p values < 0.05.

### WBC counts in the three groups (NCs versus ICs and NCs versus NICs groups)

**Figure 2:**
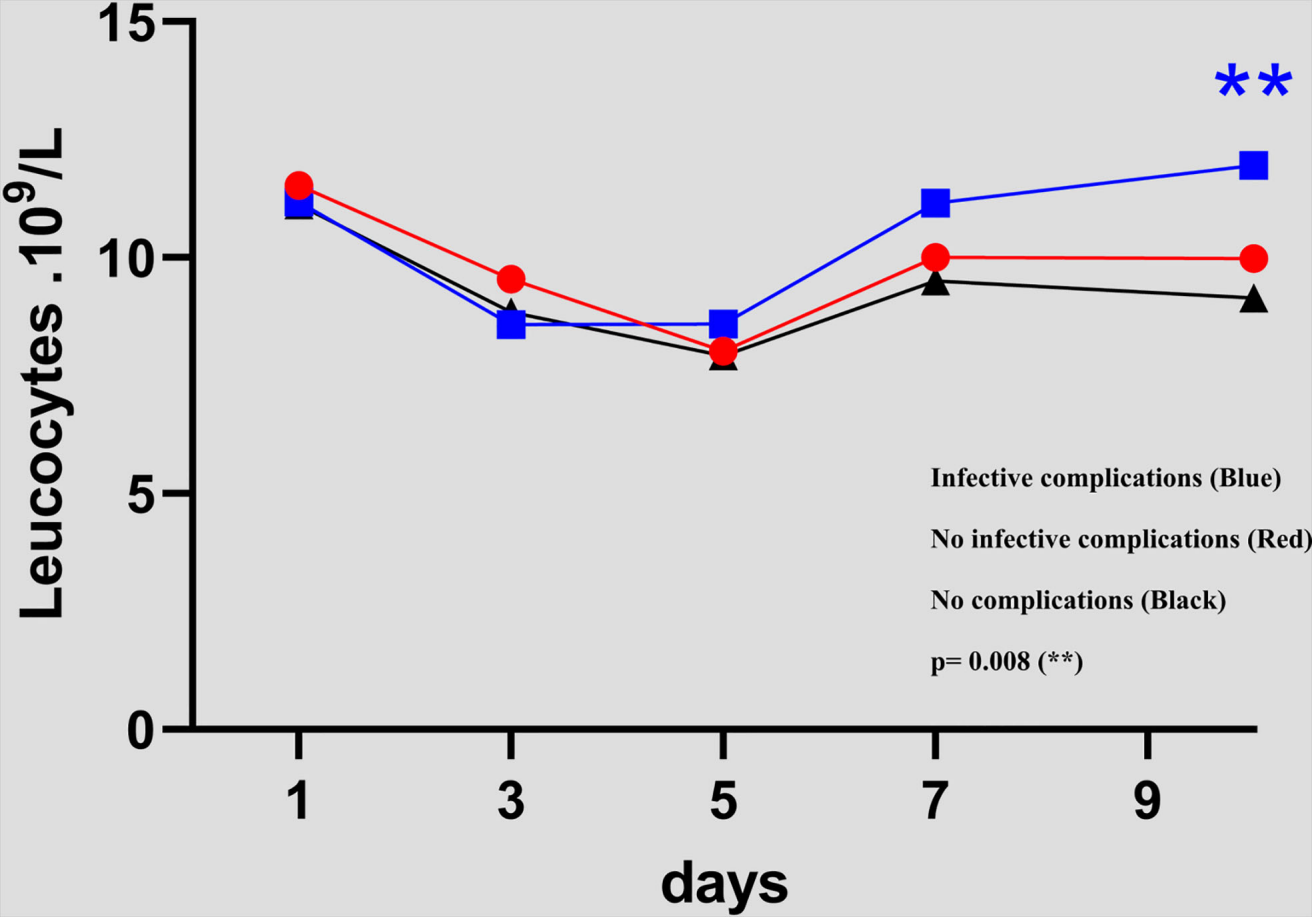
Evolution of white blood cell count between day 1 and day 10 in the 3 groups *(NCs versus ICs and NCs versus NICs groups).*.

For the three groups, the white blood cell counts decreased gradually from POD1 to POD 5, then increased until POD 10. The only significant difference between the groups with infective complications and no complications occurred at POD 10 (9.1 versus 11.9 p= 0.008). **Figure 2**

### Postoperative laboratory data and predictive value of CRP for patients with infective complications

**Figure 3:**
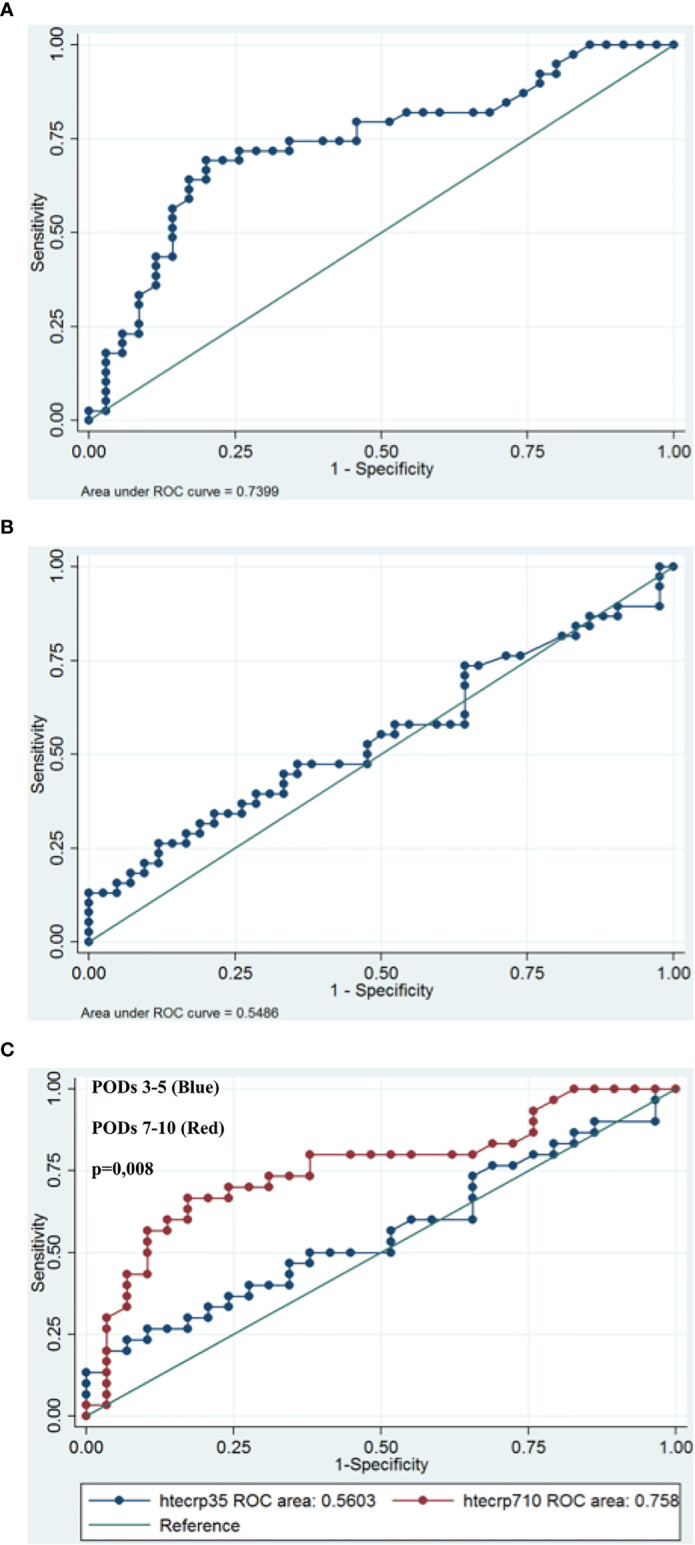
Receiver operating characteristic (ROC) curves for CRP on postoperative day (PODs) 3-5 **(A)** and PODs 7-10 **(B)** for patients with infective complications. An investigation of cut-off scores showed that the optimal CRP cut-off value was 169 mg/l on PODs 3-5 (sensitivity 26.3%; specificity 88.1%) and 62 mg/l on PODs 7-10 (sensitivity 69.2%, specificity 80%). The area under the ROC curve was 0,56 on PODs 3-5 and 0,76 on POD 7-10, p=0.0071 **(C)**.

We performed a univariate analysis of the highest CRP level in order to search for a predictive value and we divided patients into two groups: those with very early ICs (PODs 3-5) and those with ICs from the second week (PODs 7-10). We analyzed the ROC curve from PODs 3-5 and PODs 7-10. A cut-off CRP value of 169 mg/L had a sensitivity of 26.3% and a specificity of 88.1% for postoperative infective complications at PODs 3-5. A cut-off CRP value of 62 mg/L at PODs 7-10 represented the optimal cut-off (69.2% sensitivity and 80% specificity). The area under the curve (AUC) was significantly lower at PODs 3-5 than at PODs 7-10 (0.56, 95% Confidence. Interval: [0.41108-0.70961] versus 0,76, 95% Confidence Interval: [0.63086-0.88523], p=0.007). **Figures 3A–C**

Among the 53 patients with ICs, 29 patients had a CRP value higher than these cut-offs (True positive, sensibility = 55%). The percentage of patients who had CRP values above that threshold at any point and with ICS (positive predictive value) was 74%. Moreover, 17 patients (32%) with ICs had a CRP value higher than these cut-offs before the diagnosis was made by the medical team. The mean of delay between the date of “predictive CRP value” and the date of “diagnosis” was 2.9 days (Range 1 - 7). The 3 patients with anastomotic leak and 2 of the 3 patients with intrabdominal abscesses had a delay in diagnosis of 1 to 4 days.

Among 58 patients of NICs group, 10 patients without infective complication had CRP value higher than these cut-offs (False positive, 17%). The specificity and negative predictive value were 83% and 67%, respectively.

Multivariable analysis included PCI (p = 0.003), ovarian PM (p = 0.03), Pseudomyxoma and mesothelioma (p=0,003) in the final model. All three variables are demonstrated to be independent risk factors for the occurrence of IC (p value <0.05).

## Discussion

Some inflammatory markers, such as CRP and WBCs, have been used as useful tools to observe postoperative evolution and to diagnose postoperative complications after oncological surgery. We investigated the reliability of CRP and WBC values for predicting ICs after CRS and HIPEC for peritoneal metastases of diverse origins. To our knowledge, our study represents in the literature, the second work assessing the usefulness of CRP in PM from digestive and ovarian origins. These results suggested a significant association between postoperative complications after CRS and HIPEC and postoperative CRP elevation from POD3 to POD10. Moreover, the CRP cut-off value on PODs 3-5 (169 mg/l) and PODs 7-10 (62 mg/l) represented a risk factor for postoperative infective complications. The area under the curve (AUC) was significantly higher at PODs 7-10 than at PODs 5-7 (0,76 versus 0.56, p=0.007).

This study suggests that CRP cut-off may be used in clinical practice after CRS and HIPEC, specifically after POD7, before any symptoms appear. In practice, if the CRP value is higher than these cut-off values, the medical team should perform the appropriate biological or radiological exams to diagnose and treat postoperative complications earlier.

The study population encompasses the large spectrum of patients undergoing CRS and HIPEC, as our study was a consecutive series, and global postoperative complications occurred in 54.3% of the patients, which is similar than the published complication rates (3).

CRP is a nonspecific inflammatory protein synthesised by the liver and has a short half-life (~19 h) such that the serum level quickly returns to normal when patients recover (12). It is produced in response to proinflammatory cytokines that pivotal role in the amplification of the inflammatory response and can increase in many different situations, such as cancer (13), infection, inflammatory disease (14), and thrombosis. Thus, it can be tested easily at low cost and with good reliability. In digestive surgery, it can be used as a marker of postoperative complications, specifically infective complications such as anastomosis leakage after colorectal (5, 6), pancreatic (15) or oesophageal (16), bariatric (17) surgery or even infectious complications in mesh repair in ventral hernia (18).

However, the systemic inflammatory response can be secondary to HIPEC chemotherapy (19, 20). This study confirmed the conclusion of Roth et al. (19) and more recently Van Kooten et al. (21). We found a peak of postoperative inflammation after the HIPEC procedure in patients without postoperative complications in the first 3 days. Nevertheless, we did not compare the different HIPEC protocols, and this CRP increase was more significant after HIPEC with mitomycin or cisplatin. Moreover, the systemic inflammatory response after CRS can be correlated with surgical stress parameters such as blood loss, surgical dissection, open surgery (22) and operation time (23), which is particularly long in peritoneal surgery. This may explain our results at PODs 3-5 (CRP cut off = 169 mg/L).

Nevertheless, to our knowledge, this is the first study that evaluated CRP cut-off values after CRS and HIPEC in order to analyse infective/non-infective complications and early/later complications.

The value was 62 mg/L on PODs 7-10. This low value is comparable to the study of Pochhammer et al. but unusual compared to other studies, and we were expecting a higher cut-off point for patients who had HIPEC (18). For example, the CRP cut-off value was 125 mg/mL at POD4 for the detection of anastomotic leakage in colorectal surgery, for Lagoutte et al. (5), and for Ortega-Deballon et al. (24). In the literature, there are few data on the use of the CRP cut-off value after peritoneal surgery. In addition, there is heterogeneity of cut-off values and days of CRP measurement, ranging from the day of surgery to POD30. Finally, it is difficult to compare all these studies in view of the various criteria used to predict postoperative complications, such as procalcitonin, cytokines, the CRP/WBC ratio and even the CRP/albumin ratio (25, 26).

We included all complications of the use of the CRP cut-off value in clinical practice in order to analyse separately the infective and non-infective complications. For example, anastomotic leakage could induce a stronger inflammatory response than non-infective complications as pulmonary embolism (21) and may explain this CRP cut-off rate difference between the literature and our study.

Although, some of authors aimed to predict all severe postoperative complications including non-infective complications, such as Van Kooten et al. (21). However, non-infective complications rate represented 10.3% in their study, and this low rate could explain the same CRP cut-off on PODs 3-5 with these both studies (166 mg/L on POD 3 (21) versus 169 mg/L on PODs 3-5 in our study).

The more representative infectious complication in our study was urinary tract infection, which can be an explanation for the low CRP cut-off value on PODs 7-10. This high rate can be explained by the use of morphine (27); the RAAC protocol (early mobilization), which was implemented only recently in our centre; and, finally, the duration of the bladder survey that could exceed 1 week after peritonectomy of the bladder peritoneum.

The most common medical complication was acute renal failure (3.1%, 4/127), probably secondary to cisplatin. Indeed, the main side effect of cisplatin, commonly used in HIPEC (9% in this study), is nephrotoxicity (28). Nevertheless, to prevent acute renal failure, many authors use recently sodium thiosulfate during cisplatin-HIPEC (29). All respiratory complications accounted for 4.7% of cases (6/127), including pneumonia, atelectasis, and pleural effusion, which is similar to that of the literature (30). This rate can be explained by the peritonectomy of the two diaphragmatic domes and of the operating time, which regularly exceeds 10 hours after CRS and HIPEC (31, 32).

### Limitations

Several limitations to this study must be considered. Our study is limited by its retrospective, single-centre design, and a small number of subjects constituted the groups, especially with non-infective complications. Nevertheless, the study population represents the complete spectrum of patients with PM at a large-volume oncological centre and were followed every day by peritoneal surgeons and anaesthetists. Hence, these results seem to be applicable to surgical practice but need to be confirmed in prospective studies, including the use of other parameters such as the CRP-to-albumin ratio, platelet-to-lymphocyte ratio, procalcitonin, and cytokines.

## Conclusion

In conclusion, our findings suggest that routine measurement of CRP after POD3 can provide information for oncological surgeons to guide postoperative management. The CRP cut-off value on PODs 3-5 (169 mg/l) and PODs 7-10 (62 mg/l) can be useful for the early diagnosis of postoperative infectious complications after CRS and HIPEC.

## Data availability statement

The original contributions presented in the study are included in the article/**Supplementary Material**. Further inquiries can be directed to the corresponding author.

## Author contributions

AR: Study concepts and design, data acquisition, quality control of data and algorithms, data analysis and interpretation, manuscript preparation, editing and review. VD: Manuscript preparation, editing and review. EA: Quality control of data and algorithms, data analysis and interpretation. SB: Quality control of data and algorithms, data analysis and interpretation. SD: Study concepts and design, data analysis and interpretation, manuscript preparation, editing and review. AT: Study concepts and design, data acquisition, quality control of data and algorithms, data analysis and interpretation, manuscript preparation, editing and review.

## Funding

Limoges University Hospital contributed to the financing of the publication costs.

## Conflict of interest

The authors declare that the research was conducted in the absence of any commercial or financial relationships that could be construed as a potential conflict of interest.

## Publisher’s note

All claims expressed in this article are solely those of the authors and do not necessarily represent those of their affiliated organizations, or those of the publisher, the editors and the reviewers. Any product that may be evaluated in this article, or claim that may be made by its manufacturer, is not guaranteed or endorsed by the publisher.

## Supplementary material

Annex1 Flow chart.
