# Supplementary figures and images for: Predictive value of C-reactive protein levels for the early and later detection of postoperative complications after cytoreductive surgery and HIPEC
Source: Front Oncol. 2022 Oct 25;12:943522. doi: 10.3389/fonc.2022.943522 (PMC9641749; doi:10.3389/fonc.2022.943522)

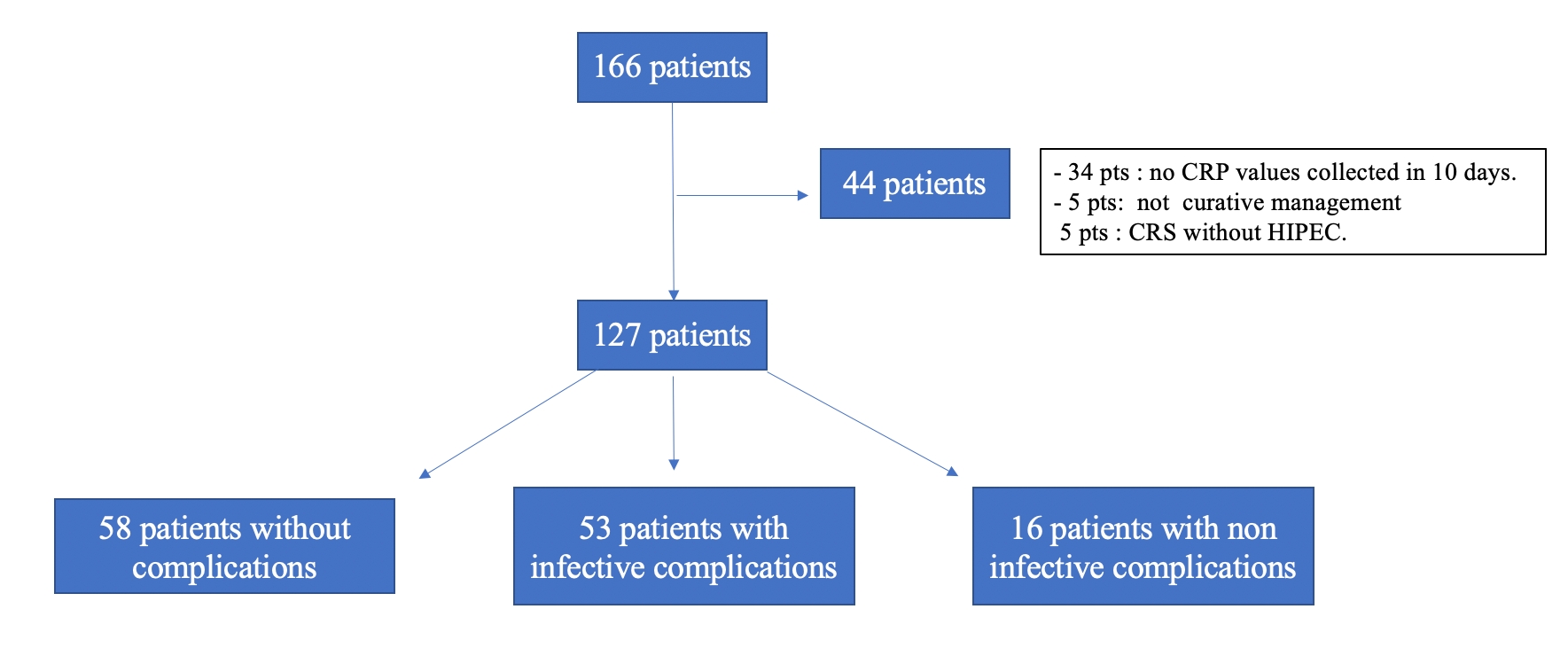

Supplement: Annex1 — Flow chart. [file Image_1.jpeg]
