# Supplementary material for: Predictive value of C-reactive protein levels for the early and later detection of postoperative complications after cytoreductive surgery and HIPEC
Source: Front Oncol. 2022 Oct 25;12:943522. doi: 10.3389/fonc.2022.943522 (PMC9641749; doi:10.3389/fonc.2022.943522)
Supplement: Supplementary file 2 [file Table_2.docx]

**Annex 2: Diagnosis of postoperative complications**

**Pneumonia:** listening to your lungs with a stethoscope to check for abnormal bubbling or crackling sounds and confirmed with Chest X-ray or CT scan or sputum culture

**Colitis:** Abdominal pain / Blood in your stool / Ongoing diarrhea and confirmed with CT Scan

**Urinary tract infection:** A strong, Persistent urge to urinate / A burning sensation when urinating / Passing frequent, Small amounts of urine / Urine that appears cloudy / Strong-smelling urine / Pelvic pain / Types of urinary tract infection and confirmed with urinalysis

**Wound abscess**: Involves the skin and subcutaneous tissues / Purulent discharge from the wound and diagnosed by the surgeon.

**Infection of central venous catheter** : fever / chills / unexplained hypotension and confirmed with positive culture of blood from a peripheral and central vein or positive culture of central catheter

**Phlebitis** : Warmth, tenderness and pain in the affected area / Redness and swelling / elevated blood level of D dimer and confirmed with venous ultrasound

**Respiratory complication** : Difficulty breathing (dyspnea) / Shortness / cough / Wheezing for breath and confirmed by anesthesit

**Acute kidney failure** : Decreased urine output / Swelling in your legs, ankles or feet / Shortness of breath and confirmed with blood test (rapidly rising levels of urea and creatinine)

**Anastomosis leakage** : Abdominal pain / Nausea and vomiting / Persistent paralytic ileus / Tachycardia / Sepsis / Shock and confirmed with CT Scan

**Intraabdominal abcess** : Abdominal pain / fever / Nausea and vomiting / Change in bowel movements and confirmed with CT Scan (whatever the size)

**Small bowel obstruction** : Abdominal pain / Nausea and vomiting and confirmed with CT Scan and confirmed with CT Scan and confirmed with CT Scan and confirmed with CT Scan and confirmed with CT Scan

**Post operative bleeding** : Abdominal pain / Tachycardia / low blood pressure / Hemorrhagic fluid in abdomianl drains and confirmed with CT Scan

**Pulmonary embolism** : Rapid or irregular heartbeat / dizziness / Excessive sweating and confirmed with CT Scan
