# Supplementary material for: Predictive value of C-reactive protein levels for the early and later detection of postoperative complications after cytoreductive surgery and HIPEC
Source: Front Oncol. 2022 Oct 25;12:943522. doi: 10.3389/fonc.2022.943522 (PMC9641749; doi:10.3389/fonc.2022.943522)

**Annex 3:** the table of sensitivity, specificity and positive negative likelihood ratios for each CRP value (on red the cut-off values).

Detailed report of Sensitivity and Specificity

------------------------------------------------------------------------------

Cutpoint Sensitivity Specificity Classified LR+ LR-

------------------------------------------------------------------------------

( >= 0 ) 100.00% 0.00% 47.50% 1.0000

( >= 16 ) 100.00% 2.38% 48.75% 1.0244 0.0000

( >= 18 ) 97.37% 2.38% 47.50% 0.9974 1.1053

( >= 20 ) 94.74% 2.38% 46.25% 0.9705 2.2105

( >= 25 ) 89.47% 2.38% 43.75% 0.9166 4.4210

( >= 26 ) 89.47% 9.52% 47.50% 0.9889 1.1053

( >= 27 ) 86.84% 9.52% 46.25% 0.9598 1.3816

( >= 28 ) 86.84% 11.90% 47.50% 0.9858 1.1053

( >= 29 ) 86.84% 14.29% 48.75% 1.0132 0.9211

( >= 31 ) 84.21% 14.29% 47.50% 0.9825 1.1053

( >= 32 ) 84.21% 16.67% 48.75% 1.0105 0.9474

( >= 33 ) 81.58% 16.67% 47.50% 0.9789 1.1053

( >= 35 ) 81.58% 19.05% 48.75% 1.0077 0.9671

( >= 37 ) 76.32% 26.19% 50.00% 1.0340 0.9043

( >= 39 ) 76.32% 28.57% 51.25% 1.0684 0.8289

( >= 40 ) 73.68% 33.33% 52.50% 1.1053 0.7895

( >= 41 ) 73.68% 35.71% 53.75% 1.1462 0.7368

( >= 42 ) 71.05% 35.71% 52.50% 1.1053 0.8105

( >= 48 ) 68.42% 35.71% 51.25% 1.0643 0.8842

( >= 49 ) 60.53% 35.71% 47.50% 0.9415 1.1053

( >= 50 ) 57.89% 35.71% 46.25% 0.9006 1.1789

( >= 52 ) 57.89% 38.10% 47.50% 0.9352 1.1053

( >= 53 ) 57.89% 40.48% 48.75% 0.9726 1.0402

( >= 55 ) 57.89% 45.24% 51.25% 1.0572 0.9307

( >= 58 ) 57.89% 47.62% 52.50% 1.1053 0.8842

( >= 59 ) 55.26% 47.62% 51.25% 1.0550 0.9395

( >= 60 ) 55.26% 50.00% 52.50% 1.1053 0.8947

( >= 62 ) 52.63% 52.38% 52.50% 1.1053 0.9043

( >= 63 ) 50.00% 52.38% 51.25% 1.0500 0.9545

( >= 65 ) 47.37% 52.38% 50.00% 0.9947 1.0048

( >= 68 ) 47.37% 57.14% 52.50% 1.1053 0.9211

( >= 70 ) 47.37% 61.90% 55.00% 1.2434 0.8502

( >= 75 ) 47.37% 64.29% 56.25% 1.3263 0.8187

( >= 76 ) 44.74% 64.29% 55.00% 1.2526 0.8596

( >= 78 ) 44.74% 66.67% 56.25% 1.3421 0.8289

( >= 82 ) 42.11% 66.67% 55.00% 1.2632 0.8684

( >= 92 ) 39.47% 66.67% 53.75% 1.1842 0.9079

( >= 94 ) 39.47% 69.05% 55.00% 1.2753 0.8766

( >= 95 ) 39.47% 71.43% 56.25% 1.3816 0.8474

( >= 96 ) 36.84% 71.43% 55.00% 1.2895 0.8842

( >= 101 ) 36.84% 73.81% 56.25% 1.4067 0.8557

( >= 111 ) 34.21% 73.81% 55.00% 1.3062 0.8913

( >= 119 ) 34.21% 76.19% 56.25% 1.4368 0.8635

( >= 126 ) 34.21% 78.57% 57.50% 1.5965 0.8373

( >= 132 ) 31.58% 78.57% 56.25% 1.4737 0.8708

( >= 136 ) 31.58% 80.95% 57.50% 1.6579 0.8452

( >= 153 ) 28.95% 80.95% 56.25% 1.5197 0.8777

( >= 162 ) 28.95% 83.33% 57.50% 1.7368 0.8526

( >= 165 ) 26.32% 83.33% 56.25% 1.5789 0.8842

( >= 168 ) 26.32% 85.71% 57.50% 1.8421 0.8596

**( >= 169 ) 26.32% 88.10% 58.75% 2.2105 0.8364**

( >= 176 ) 23.68% 88.10% 57.50% 1.9895 0.8663

( >= 182 ) 21.05% 88.10% 56.25% 1.7684 0.8962

( >= 184 ) 21.05% 90.48% 57.50% 2.2105 0.8726

( >= 194 ) 18.42% 90.48% 56.25% 1.9342 0.9017

( >= 198 ) 18.42% 92.86% 57.50% 2.5789 0.8785

( >= 226 ) 15.79% 92.86% 56.25% 2.2105 0.9069

( >= 230 ) 15.79% 95.24% 57.50% 3.3158 0.8842

( >= 236 ) 13.16% 95.24% 56.25% 2.7632 0.9118

( >= 239 ) 13.16% 97.62% 57.50% 5.5263 0.8896

( >= 241 ) 13.16% 100.00% 58.73% 0.8684

( >= 244 ) 10.53% 100.00% 57.50% 0.8947

( >= 291 ) 7.89% 100.00% 56.25% 0.9211

( >= 292 ) 5.26% 100.00% 55.00% 0.9474

( >= 299 ) 2.63% 100.00% 53.75% 0.9737

( > 299 ) 0.00% 100.00% 52.50% 1.0000

------------------------------------------------------------------------------

ROC -Asymptotic Normal--

Obs Area Std. Err. [95% Conf. Interval]

--------------------------------------------------------

**80 0.5486 0.0660 0.41911 0.67800**

**
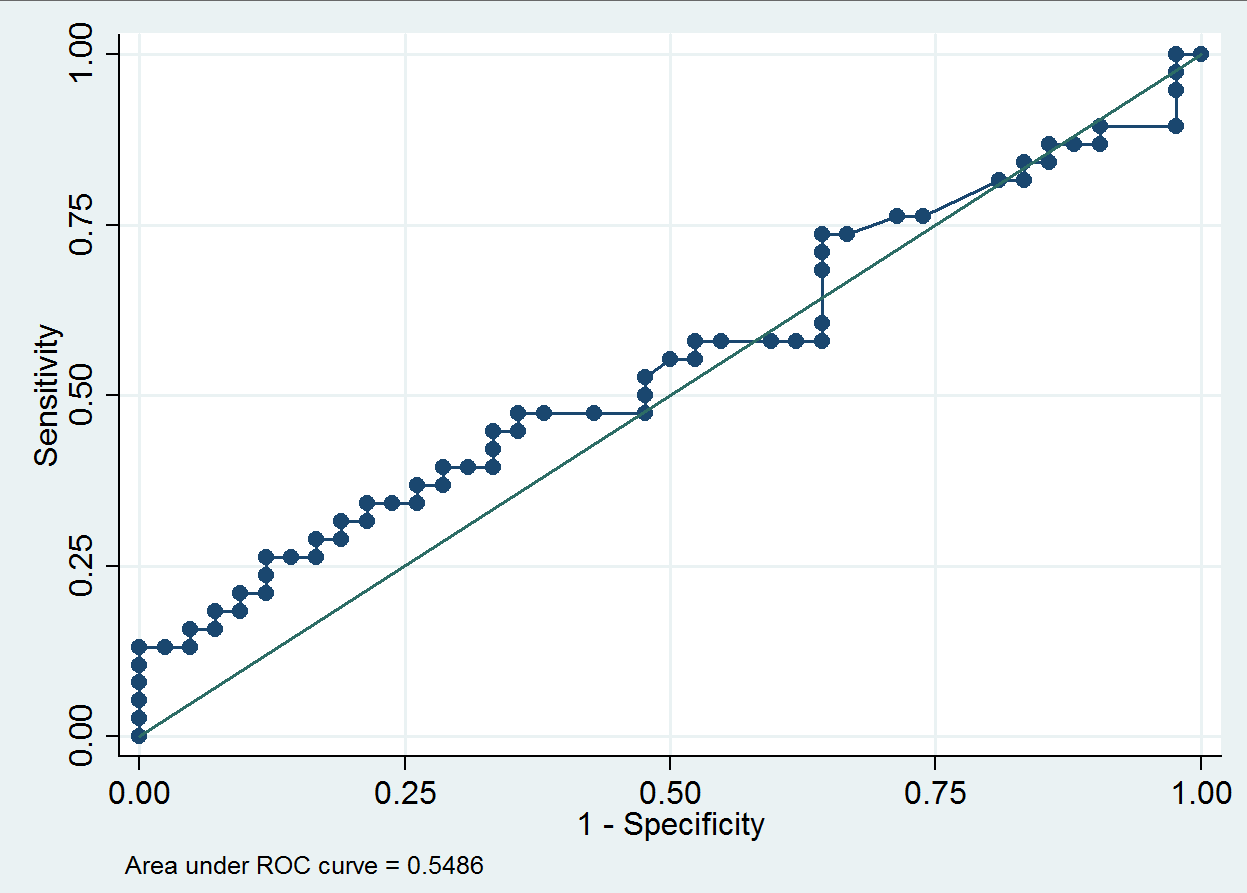
**

.

roctab complications htecrp J7-10, detail graph summary

Detailed report of Sensitivity and Specificity

------------------------------------------------------------------------------

Correctly

Cutpoint Sensitivity Specificity Classified LR+ LR-

------------------------------------------------------------------------------

( >= 0 ) 100.00% 0.00% 52.70% 1.0000

( >= 2 ) 100.00% 2.86% 54.05% 1.0294 0.0000

( >= 3 ) 100.00% 5.71% 55.41% 1.0606 0.0000

( >= 5 ) 100.00% 8.57% 56.76% 1.0937 0.0000

( >= 6 ) 100.00% 11.43% 58.11% 1.1290 0.0000

( >= 11 ) 100.00% 14.29% 59.46% 1.1667 0.0000

( >= 13 ) 97.44% 17.14% 59.46% 1.1760 0.1496

( >= 14 ) 94.87% 20.00% 59.46% 1.1859 0.2564

( >= 17 ) 92.31% 20.00% 58.11% 1.1538 0.3846

( >= 21 ) 92.31% 22.86% 59.46% 1.1966 0.3365

( >= 22 ) 89.74% 22.86% 58.11% 1.1633 0.4487

( >= 23 ) 87.18% 25.71% 58.11% 1.1736 0.4986

( >= 27 ) 84.62% 28.57% 58.11% 1.1846 0.5385

( >= 32 ) 82.05% 31.43% 58.11% 1.1966 0.5711

( >= 35 ) 82.05% 34.29% 59.46% 1.2486 0.5235

( >= 37 ) 82.05% 40.00% 62.16% 1.3675 0.4487

( >= 40 ) 82.05% 42.86% 63.51% 1.4359 0.4188

( >= 41 ) 82.05% 45.71% 64.86% 1.5115 0.3926

( >= 42 ) 79.49% 48.57% 64.86% 1.5456 0.4223

( >= 43 ) 79.49% 54.29% 67.57% 1.7388 0.3779

( >= 44 ) 74.36% 54.29% 64.86% 1.6266 0.4723

( >= 47 ) 74.36% 57.14% 66.22% 1.7350 0.4487

( >= 49 ) 74.36% 60.00% 67.57% 1.8590 0.4274

( >= 51 ) 74.36% 65.71% 70.27% 2.1688 0.3902

( >= 52 ) 71.79% 65.71% 68.92% 2.0940 0.4292

( >= 53 ) 71.79% 68.57% 70.27% 2.2844 0.4113

( >= 57 ) 71.79% 71.43% 71.62% 2.5128 0.3949

( >= 58 ) 71.79% 74.29% 72.97% 2.7920 0.3797

( >= 59 ) 69.23% 74.29% 71.62% 2.6923 0.4142

( >= 61 ) 69.23% 77.14% 72.97% 3.0288 0.3989

**( >= 62 ) 69.23% 80.00% 74.32% 3.4615 0.3846**

( >= 73 ) 66.67% 80.00% 72.97% 3.3333 0.4167

( >= 76 ) 64.10% 80.00% 71.62% 3.2051 0.4487

( >= 81 ) 64.10% 82.86% 72.97% 3.7393 0.4332

( >= 84 ) 61.54% 82.86% 71.62% 3.5897 0.4642

( >= 88 ) 58.97% 82.86% 70.27% 3.4402 0.4951

( >= 91 ) 56.41% 85.71% 70.27% 3.9487 0.5085

( >= 97 ) 53.85% 85.71% 68.92% 3.7692 0.5385

( >= 98 ) 51.28% 85.71% 67.57% 3.5897 0.5684

( >= 124 ) 48.72% 85.71% 66.22% 3.4103 0.5983

( >= 128 ) 43.59% 85.71% 63.51% 3.0513 0.6581

( >= 129 ) 43.59% 88.57% 64.86% 3.8141 0.6369

( >= 131 ) 41.03% 88.57% 63.51% 3.5897 0.6658

( >= 132 ) 38.46% 88.57% 62.16% 3.3654 0.6948

( >= 134 ) 35.90% 88.57% 60.81% 3.1410 0.7237

( >= 141 ) 33.33% 91.43% 60.81% 3.8889 0.7292

( >= 148 ) 30.77% 91.43% 59.46% 3.5897 0.7572

( >= 160 ) 25.64% 91.43% 56.76% 2.9915 0.8133

( >= 164 ) 23.08% 91.43% 55.41% 2.6923 0.8413

( >= 186 ) 23.08% 94.29% 56.76% 4.0385 0.8159

( >= 192 ) 20.51% 94.29% 55.41% 3.5897 0.8430

( >= 193 ) 17.95% 94.29% 54.05% 3.1410 0.8702

( >= 219 ) 17.95% 97.14% 55.41% 6.2821 0.8446

( >= 252 ) 15.38% 97.14% 54.05% 5.3846 0.8710

( >= 269 ) 12.82% 97.14% 52.70% 4.4872 0.8974

( >= 273 ) 10.26% 97.14% 51.35% 3.5897 0.9238

( >= 288 ) 7.69% 97.14% 50.00% 2.6923 0.9502

( >= 307 ) 5.13% 97.14% 48.65% 1.7949 0.9766

( >= 330 ) 2.56% 97.14% 47.30% 0.8974 1.0030

( >= 497 ) 2.56% 100.00% 48.65% 0.9744

( > 497 ) 0.00% 100.00% 47.30% 1.0000

------------------------------------------------------------------------------

ROC -Asymptotic Normal--

Obs Area Std. Err. [95% Conf. Interval]

--------------------------------------------------------

**74 0.7399 0.0594 0.62360 0.85625**

**
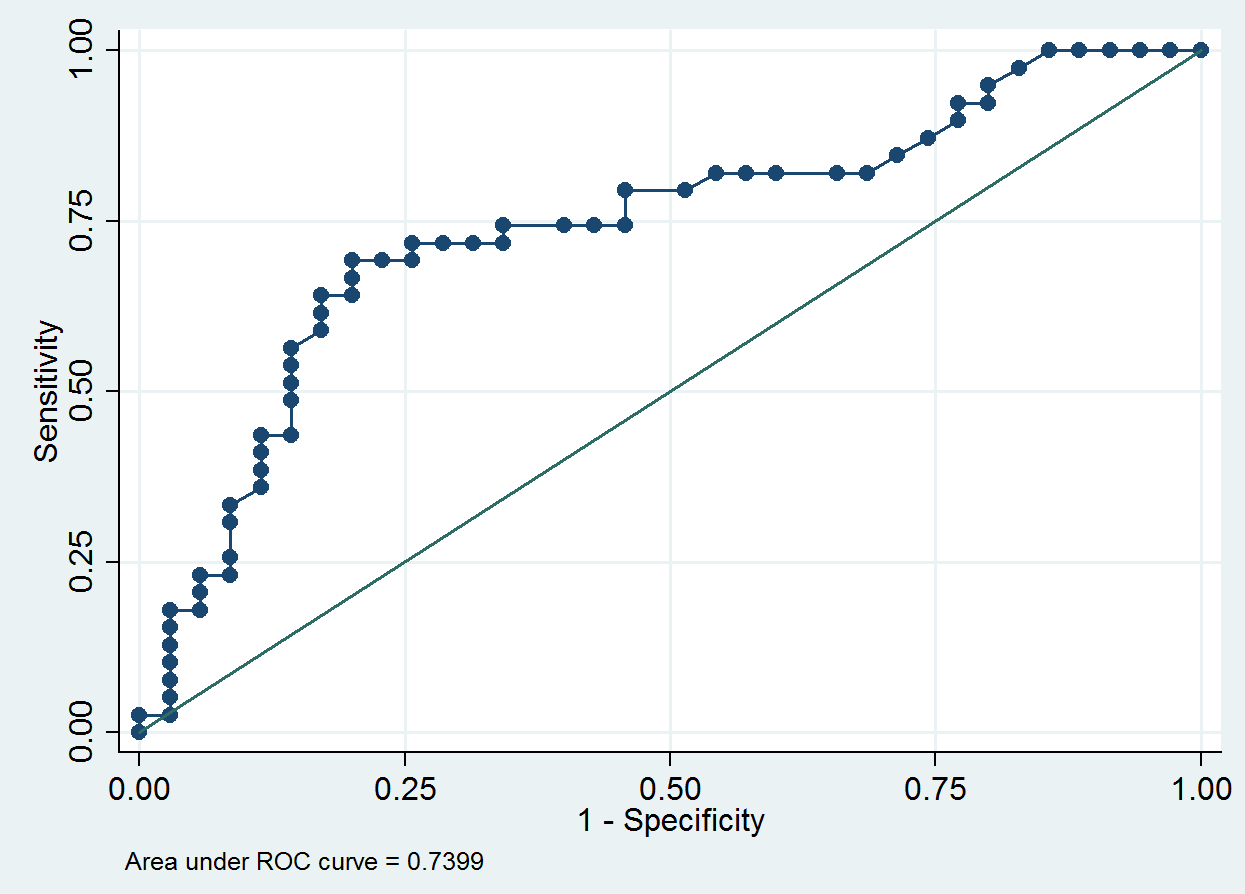
**

. roccomp v29 htecrp35 htecrp710, graph summary

ROC -Asymptotic Normal--

Obs Area Std. Err. [95% Conf. Interval]

-------------------------------------------------------------------------

htecrp35 59 0.5603 0.0762 0.41108 0.70961

htecrp710 59 0.7580 0.0649 0.63086 0.88523

-------------------------------------------------------------------------

Ho: area(htecrp35) = area(htecrp710)

chi2(1) = 7.24 **Prob>chi2 = 0.0071**


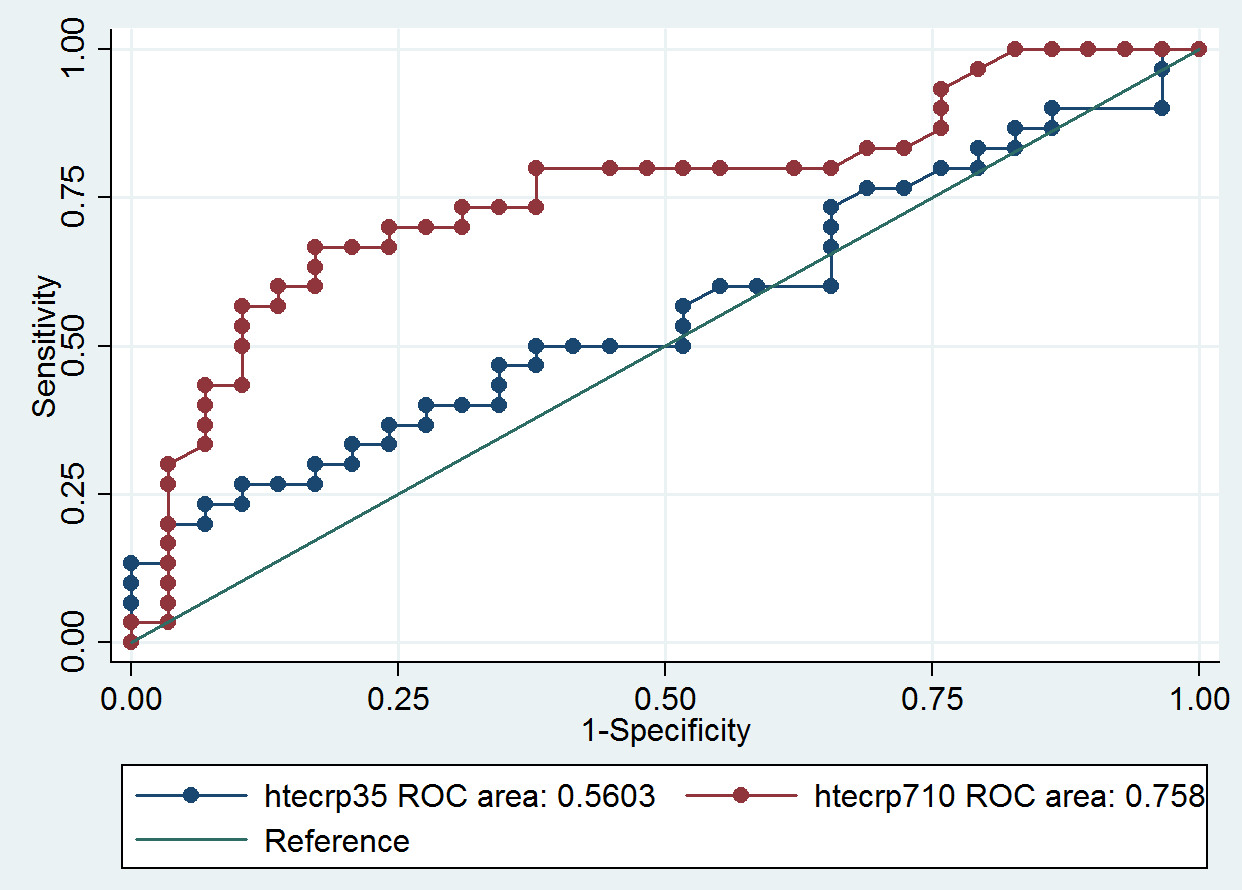


Détail J3-J5

. roctab complications j3j5, detail graph summary

Detailed report of Sensitivity and Specificity

------------------------------------------------------------------------------

Correctly

Cutpoint Sensitivity Specificity Classified LR+ LR-

------------------------------------------------------------------------------

( >= 0 ) 100.00% 0.00% 50.85% 1.0000

( >= 18 ) 100.00% 3.45% 52.54% 1.0357 0.0000

( >= 20 ) 96.67% 3.45% 50.85% 1.0012 0.9667

( >= 25 ) 90.00% 3.45% 47.46% 0.9321 2.9000

( >= 26 ) 90.00% 13.79% 52.54% 1.0440 0.7250

( >= 27 ) 86.67% 13.79% 50.85% 1.0053 0.9667

( >= 29 ) 86.67% 17.24% 52.54% 1.0472 0.7733

( >= 31 ) 83.33% 17.24% 50.85% 1.0069 0.9667

( >= 32 ) 83.33% 20.69% 52.54% 1.0507 0.8056

( >= 33 ) 80.00% 20.69% 50.85% 1.0087 0.9667

( >= 35 ) 80.00% 24.14% 52.54% 1.0545 0.8286

( >= 37 ) 76.67% 27.59% 52.54% 1.0587 0.8458

( >= 39 ) 76.67% 31.03% 54.24% 1.1117 0.7519

( >= 41 ) 73.33% 34.48% 54.24% 1.1193 0.7733

( >= 42 ) 70.00% 34.48% 52.54% 1.0684 0.8700

( >= 48 ) 66.67% 34.48% 50.85% 1.0175 0.9667

( >= 53 ) 60.00% 34.48% 47.46% 0.9158 1.1600

( >= 59 ) 60.00% 41.38% 50.85% 1.0235 0.9667

( >= 60 ) 60.00% 44.83% 52.54% 1.0875 0.8923

( >= 62 ) 56.67% 48.28% 52.54% 1.0956 0.8976

( >= 63 ) 53.33% 48.28% 50.85% 1.0311 0.9667

( >= 65 ) 50.00% 48.28% 49.15% 0.9667 1.0357

( >= 68 ) 50.00% 55.17% 52.54% 1.1154 0.9063

( >= 70 ) 50.00% 58.62% 54.24% 1.2083 0.8529

( >= 75 ) 50.00% 62.07% 55.93% 1.3182 0.8056

( >= 76 ) 46.67% 62.07% 54.24% 1.2303 0.8593

( >= 78 ) 46.67% 65.52% 55.93% 1.3533 0.8140

( >= 82 ) 43.33% 65.52% 54.24% 1.2567 0.8649

( >= 92 ) 40.00% 65.52% 52.54% 1.1600 0.9158

( >= 94 ) 40.00% 68.97% 54.24% 1.2889 0.8700

( >= 95 ) 40.00% 72.41% 55.93% 1.4500 0.8286

( >= 96 ) 36.67% 72.41% 54.24% 1.3292 0.8746

( >= 101 ) 36.67% 75.86% 55.93% 1.5190 0.8348

( >= 132 ) 33.33% 75.86% 54.24% 1.3810 0.8788

( >= 136 ) 33.33% 79.31% 55.93% 1.6111 0.8406

( >= 153 ) 30.00% 79.31% 54.24% 1.4500 0.8826

( >= 162 ) 30.00% 82.76% 55.93% 1.7400 0.8458

( >= 165 ) 26.67% 82.76% 54.24% 1.5467 0.8861

( >= 168 ) 26.67% 86.21% 55.93% 1.9333 0.8507

**( >= 176 ) 26.67% 89.66% 57.63% 2.5778 0.8179**

( >= 182 ) 23.33% 89.66% 55.93% 2.2556 0.8551

( >= 184 ) 23.33% 93.10% 57.62% 3.3833 0.8235

( >= 194 ) 20.00% 93.10% 55.93% 2.9000 0.8593

( >= 198 ) 20.00% 96.55% 57.62% 5.8000 0.8286

( >= 230 ) 16.67% 96.55% 55.93% 4.8333 0.8631

( >= 236 ) 13.33% 96.55% 54.24% 3.8667 0.8976

( >= 244 ) 13.33% 100.00% 55.93% 0.8667

( >= 291 ) 10.00% 100.00% 54.24% 0.9000

( >= 292 ) 6.67% 100.00% 52.54% 0.9333

( >= 299 ) 3.33% 100.00% 50.85% 0.9667

( > 299 ) 0.00% 100.00% 49.15% 1.0000

------------------------------------------------------------------------------

ROC -Asymptotic Normal--

Obs Area Std. Err. [95% Conf. Interval]

--------------------------------------------------------

**59 0.5603 0.0762 0.41108 0.70961**


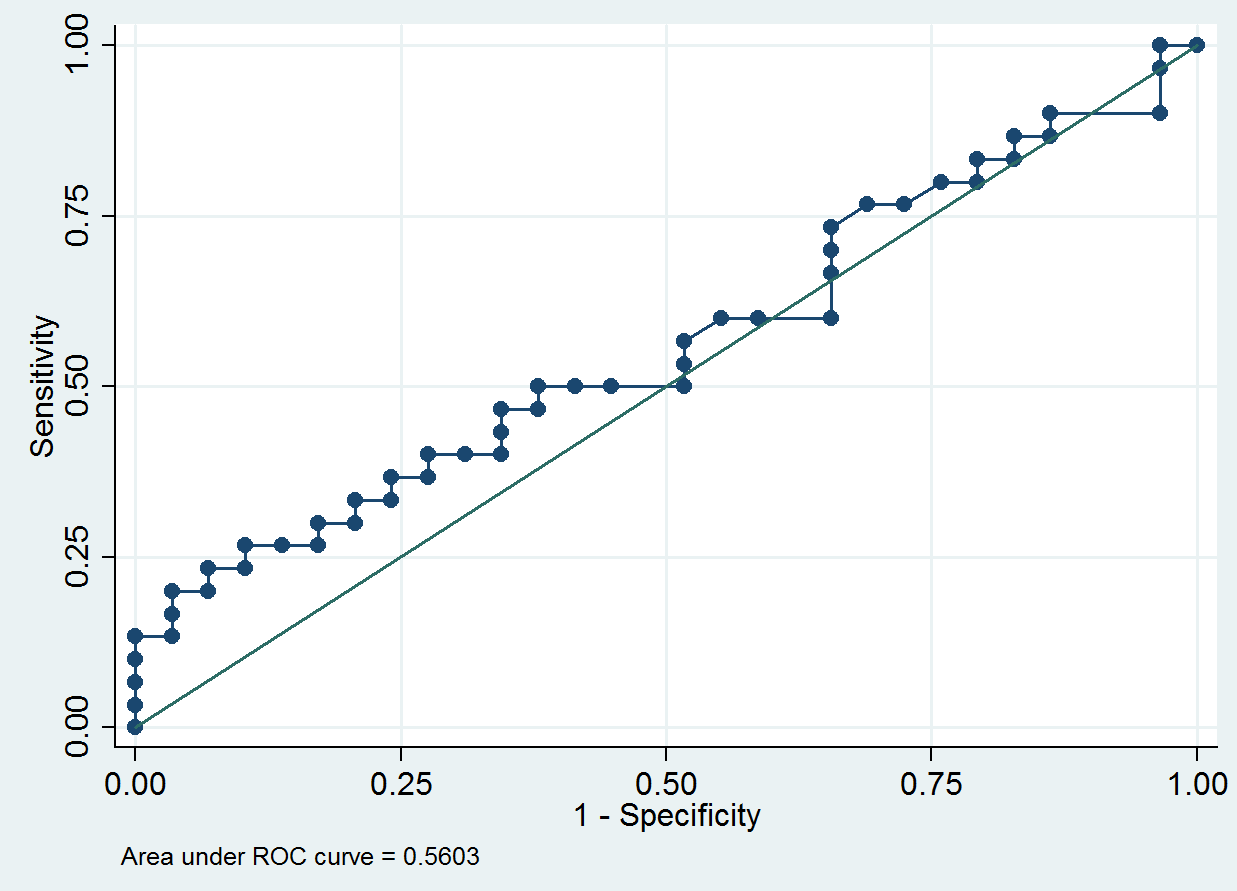


Détails J7-J10

. roctab complications j7j10, detail graph summary

Detailed report of Sensitivity and Specificity

------------------------------------------------------------------------------

Correctly

Cutpoint Sensitivity Specificity Classified LR+ LR-

------------------------------------------------------------------------------

( >= 0 ) 100.00% 0.00% 50.85% 1.0000

( >= 2 ) 100.00% 3.45% 52.54% 1.0357 0.0000

( >= 3 ) 100.00% 6.90% 54.24% 1.0741 0.0000

( >= 5 ) 100.00% 10.34% 55.93% 1.1154 0.0000

( >= 6 ) 100.00% 13.79% 57.63% 1.1600 0.0000

( >= 11 ) 100.00% 17.24% 59.32% 1.2083 0.0000

( >= 13 ) 96.67% 20.69% 59.32% 1.2188 0.1611

( >= 14 ) 93.33% 24.14% 59.32% 1.2303 0.2762

( >= 21 ) 90.00% 24.14% 57.63% 1.1864 0.4143

( >= 22 ) 86.67% 24.14% 55.93% 1.1424 0.5524

( >= 23 ) 83.33% 27.59% 55.93% 1.1508 0.6042

( >= 27 ) 83.33% 31.03% 57.63% 1.2083 0.5370

( >= 32 ) 80.00% 34.48% 57.63% 1.2211 0.5800

( >= 35 ) 80.00% 37.93% 59.32% 1.2889 0.5273

( >= 37 ) 80.00% 44.83% 62.71% 1.4500 0.4462

( >= 40 ) 80.00% 48.28% 64.41% 1.5467 0.4143

( >= 41 ) 80.00% 51.72% 66.10% 1.6571 0.3867

( >= 42 ) 80.00% 55.17% 67.80% 1.7846 0.3625

( >= 43 ) 80.00% 62.07% 71.19% 2.1091 0.3222

( >= 44 ) 73.33% 62.07% 67.80% 1.9333 0.4296

( >= 47 ) 73.33% 65.52% 69.49% 2.1267 0.4070

( >= 51 ) 73.33% 68.97% 71.19% 2.3630 0.3867

( >= 53 ) 70.00% 68.97% 69.49% 2.2556 0.4350

( >= 57 ) 70.00% 72.41% 71.19% 2.5375 0.4143

( >= 58 ) 70.00% 75.86% 72.88% 2.9000 0.3955

( >= 59 ) 66.67% 75.86% 71.19% 2.7619 0.4394

( >= 61 ) 66.67% 79.31% 72.88% 3.2222 0.4203

**( >= 62 ) 66.67% 82.76% 74.58% 3.8667 0.4028**

( >= 73 ) 63.33% 82.76% 72.88% 3.6733 0.4431

( >= 76 ) 60.00% 82.76% 71.19% 3.4800 0.4833

( >= 84 ) 60.00% 86.21% 72.88% 4.3500 0.4640

( >= 88 ) 56.67% 86.21% 71.19% 4.1083 0.5027

( >= 97 ) 56.67% 89.66% 72.88% 5.4778 0.4833

( >= 98 ) 53.33% 89.66% 71.19% 5.1556 0.5205

( >= 124 ) 50.00% 89.66% 69.49% 4.8333 0.5577

( >= 128 ) 43.33% 89.66% 66.10% 4.1889 0.6321

( >= 129 ) 43.33% 93.10% 67.80% 6.2833 0.6086

( >= 131 ) 40.00% 93.10% 66.10% 5.8000 0.6444

( >= 132 ) 36.67% 93.10% 64.41% 5.3167 0.6802

( >= 134 ) 33.33% 93.10% 62.71% 4.8333 0.7160

( >= 141 ) 30.00% 96.55% 62.71% 8.7000 0.7250

( >= 148 ) 26.67% 96.55% 61.02% 7.7333 0.7595

( >= 160 ) 20.00% 96.55% 57.63% 5.8000 0.8286

( >= 186 ) 16.67% 96.55% 55.93% 4.8333 0.8631

( >= 219 ) 13.33% 96.55% 54.24% 3.8667 0.8976

( >= 252 ) 10.00% 96.55% 52.54% 2.9000 0.9321

( >= 307 ) 6.67% 96.55% 50.85% 1.9333 0.9667

( >= 330 ) 3.33% 96.55% 49.15% 0.9667 1.0012

( >= 497 ) 3.33% 100.00% 50.85% 0.9667

( > 497 ) 0.00% 100.00% 49.15% 1.0000

------------------------------------------------------------------------------

ROC -Asymptotic Normal--

Obs Area Std. Err. [95% Conf. Interval]

--------------------------------------------------------

**59 0.7580 0.0649 0.63086 0.88523**


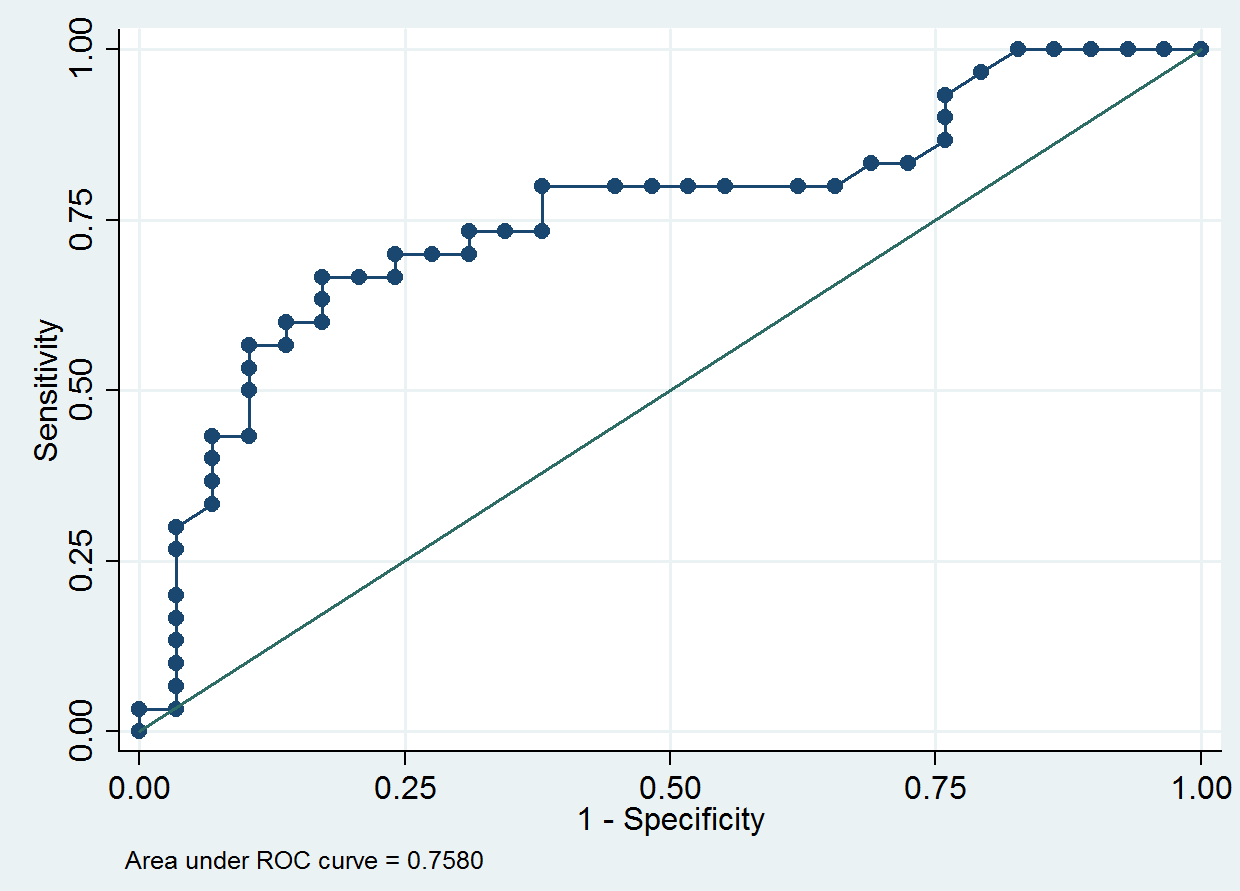

Supplement: Supplementary file 3 [file Table_3.docx]
